# Randomized phase II study of gemcitabine and S-1 combination therapy versus gemcitabine and nanoparticle albumin-bound paclitaxel combination therapy as neoadjuvant chemotherapy for resectable/borderline resectable pancreatic ductal adenocarcinoma (PDAC-GS/GA-rP2, CSGO-HBP-015)

**DOI:** 10.1186/s13063-021-05541-w

**Published:** 2021-08-26

**Authors:** Daisaku Yamada, Shogo Kobayashi, Hidenori Takahashi, Hirofumi Akita, Terumasa Yamada, Tadafumi Asaoka, Junzo Shimizu, Yutaka Takeda, Shigekazu Yokoyama, Masanori Tsujie, Akira Tomokuni, Masahiro Tanemura, Osakuni Morimoto, Masahiro Murakami, Yongkook Kim, Shin Nakahira, Naoki Hama, Keishi Sugimoto, Kazuhiko Hashimoto, Yuichiro Doki, Hidetoshi Eguchi

**Affiliations:** 1 Department of Gastroenterological Surgery, Graduate School of Medicine, Osaka University, Yamadaoka 2-2-E2, Suita, Osaka, 565-0871 Japan; 2 Department of Gastroenterological Surgery, Osaka International Cancer Institute, Osaka, Japan; 3 Department of Surgery, Higashiosaka City Medical Center, Higashiōsaka, Japan; 4 Department of Gastroenterological Surgery, Osaka Police Hospital, Osaka, Japan; 5 Department of Gastroenterological Surgery, Toyonaka Municipal Hospital, Toyonaka, Japan; 6 Department of Gastroenterological Surgery, Kansai Rosai Hospital, Amagasaki, Japan; 7 Department of Gastroenterological Surgery, Hyogo Prefectural Nishinomiya Hospital, Nishinomiya, Japan; 8 Department of Gastroenterological Surgery, Osaka Rosai Hospital, Sakai, Japan; 9 Department of Gastroenterological Surgery, Osaka General Medical Center, Osaka, Japan; 10 Department of Gastroenterological Surgery, Rinku General Medical Center, Izumisano, Japan; 11 Department of Surgery, Japan Community Health Care Organization Osaka Hospital, Osaka, Japan; 12 Department of Gastroenterological Surgery, Itami City Hospital, Itami, Japan; 13 Department of Surgery, Kaizuka City Hospital, Kaizuka, Japan; 14 Department of Surgery, Sakai City Medical Center, Sakai, Japan; 15 Department of Surgery, Ikeda City Hospital, Ikeda, Japan; 16 Department of Surgery, Minoh City Hospital, Minoh, Japan; 17 Department of Surgery, Kindai University Nara Hospital, Ikoma, Japan

**Keywords:** Pancreatic cancer, Neoadjuvant chemotherapy, Gemcitabine plus S-1, Gemcitabine plus nab-paclitaxel

## Abstract

**Background:**

Pancreatic ductal adenocarcinoma (PDAC) is a lethal disease, and multimodal strategies, such as surgery plus neoadjuvant chemotherapy (NAC)/adjuvant chemotherapy, have been attempted to improve survival in patients with localized PDAC. To date, there is one prospective study providing evidence for the superiority of a neoadjuvant strategy over upfront surgery for localized PDAC. However, which NAC regimen is optimal remains unclear.

**Methods:**

A randomized, exploratory trial is performed to examine the clinical benefits of two chemotherapy regimens, gemcitabine plus S-1 (GS) and gemcitabine plus nab-paclitaxel (GA), as NAC for patients with planned PDAC resection. Patients are enrolled after the diagnosis of resectable or borderline resectable PDAC. They are randomly assigned to either NAC regimen. Adjuvant chemotherapy after curative resection is highly recommended for 6 months in both arms. The primary endpoint is tumor progression-free survival time, and secondary endpoints include the rate of curative resection, the completion rate of protocol therapy, the recurrence type, the overall survival time, and safety. The target sample size is set as at least 100.

**Discussion:**

This study is the first randomized phase II study comparing GS combination therapy with GA combination therapy as NAC for localized pancreatic cancer.

**Trial registration:**

UMIN Clinical Trials Registry UMIN000021484. This trial began in April 2016.

## Administrative information

Note: the numbers in curly brackets in this protocol refer to SPIRIT checklist item numbers. The order of the items has been modified to group similar items (see http://www.equator-network.org/reporting-guidelines/spirit-2013-statement-defining-standard-protocol-items-for-clinical-trials/).

**Table:** 

**Title {1}**	**Randomized phase II study of gemcitabine and S-1 combination therapy versus gemcitabine and nanoparticle albumin-bound paclitaxel combination therapy as neoadjuvant chemotherapy for resectable/borderline resectable pancreatic ductal adenocarcinoma (PDAC-GS/GA-rP2, CSGO-HBP-015)**
**Trial registration {2a}.**	UMIN000021484
**Protocol version {3}**	Version 4.0
**Funding {4}**	This research received no specific grant from any funding agency in the public, commercial or not-for-profit sectors.
**Author details {5a}**	1) 1)Department of Gastroenterological Surgery, Graduate School of Medicine, Osaka University 2) 2)Department of Gastroenterological Surgery, Osaka International Cancer Institute 3) 3)Department of Surgery, Higashiosaka City Medical Center 4) 4)Department of Gastroenterological Surgery, Osaka Police Hospital 5) 5)Department of Gastroenterological Surgery, Toyonaka Municipal Hospital 6) 6)Department of Gastroenterological Surgery, Kansai Rosai Hospital 7) 7)Department of Gastroenterological Surgery, Hyogo Prefectural Nishinomiya Hospital 8) 8)Department of Gastroenterological Surgery, Osaka Rosai Hospital 9) 9)Department of Gastroenterological Surgery, Osaka General Medical Center 10) 10)Department of Gastroenterological Surgery, Rinku General Medical Center 11) 11)Department of Surgery, Japan Community Health Care Organization Osaka Hospital 12) 12)Department of Gastroenterological Surgery, Itami City Hospital 13) 13Department of Surgery, Kaizuka City Hospital 14) 14)Department of Surgery, Sakai City Medical Center 15) 15)Department of Surgery, Ikeda City Hospital 16) 16)Department of Surgery, Minoh City Hospital 17) 17)Department of Surgery, Kindai University Nara Hospital
**Name and contact information for the trial sponsor {5b}**	Hidetoshi Eguchi
**Role of sponsor {5c}**	Hidetoshi Eguchi made substantial contributions to the conception and design of the study; the collection, management, analysis, and interpretation of the data; the writing of the report; and the decision to submit the report for publication, including whether they will have ultimate authority over any of these activities.

## Background and rationale {6a}

Pancreatic ductal adenocarcinoma (PDAC) is a lethal disease because tumor cells have a tendency to spread to the surrounding areas and/or distant organs, allowing PDAC to become a systemic disease from an early stage [1, 2]. Although surgery is the most important treatment for PDAC, the surgery alone strategy provides the minimum survival benefit in the majority of patients with localized PDAC [i.e., resectable (R) or borderline resectable (BR) stage in the National Comprehensive Cancer Network (NCCN) classification 2020] [3, 4]; thus, multimodal strategies, including surgery plus pre/postoperative therapies (i.e., neoadjuvant chemotherapy (NAC)/adjuvant chemotherapy), have been attempted to improve the surgical outcomes of patients with R/BR-PDAC [3–12]. First, the clinical benefit of adjuvant therapy was demonstrated. Gemcitabine (GEM) adjuvant therapy prolongs overall survival (OS), with a 5-year survival rate of 23–24% for PDAC patients undergoing curative resection [3, 13]. S-1 adjuvant chemotherapy significantly prolonged the OS of patients after surgery for PDAC compared with GEM adjuvant chemotherapy in Japanese patients, and adjuvant chemotherapy with S-1 is now the standard of care for curatively resected PDAC in Japan, with a 5-year survival rate of 44.1% in the S-1 group [4].

To date, there is one prospective study suggesting the superiority of a neoadjuvant strategy over upfront surgery for R/BR-PDAC [14], and previous reports of trials for patients with localized PDAC have suggested increased OS, supporting the benefits [9, 11]. The theoretical reasons for the demonstrated clinical benefits were assumed to be mainly the following 3 clinical standpoints: (1) early delivery of systemic therapy for almost all patients intended for treatment, (2) high tolerance of multiagent regimens by patients before undergoing surgery, and (3) a higher negative margin resection rate. However, the optimal NAC regimen for patients with R/BR-PDAC remains unclear.

This phase II trial was designed to examine the efficacy and safety of 2 regimens, gemcitabine plus S-1 (GS) and gemcitabine plus nab-paclitaxel (GA), as NAC in patients with R/BR-PDAC. The rationale behind the GS regimen was based on phase II and subsequent phase III trials for R/BR-PDAC in which NAC-GS demonstrated clinical advantages over upfront surgery with acceptable feasibility in Japan [14–16]. Thus, NAC-GS is now assumed to be a standard NAC regimen for R/BR-PDAC in Japan. The rationale behind the GA regimen was based on a phase III trial that showed a higher objective response rate (GA vs GEM, 23% vs 7%, *P* < 0.001) for GA therapy than for GEM monotherapy [17]. Likewise, GS therapy showed a higher objective response rate (29% vs 13%, *P* < 0.001) than gemcitabine monotherapy [18], and we assumed that either treatment regimen can be performed in combination with standard NAC treatments.

A nationwide survey suggested that neoadjuvant treatment might not worsen perioperative outcomes or might increase the chance for curative surgery [19]. Accordingly, it was necessary to confirm the resection rate and safety of both chemotherapy regimens in this study. The main objective of this trial was to investigate the progression-free survival (PFS, as a surrogate outcome of OS) of patients treated with either chemotherapy regimen as NAC for potentially resectable PDAC by intention-to-treat analysis.

### Objectives {7}

The primary objective of this study was to investigate the efficacy and safety of both NAC regimens, GS and GA, in patients with planned PDAC resection. The efficacy is evaluated by (1) PFS, (2) OS, (3) the curative resection rate, (4) recurrence type (if developed), and (4) radiological/histological responses. Safety is evaluated by (1) adverse events and (2) dose intensity.

### Trial design {8}

**Fig. 1:**
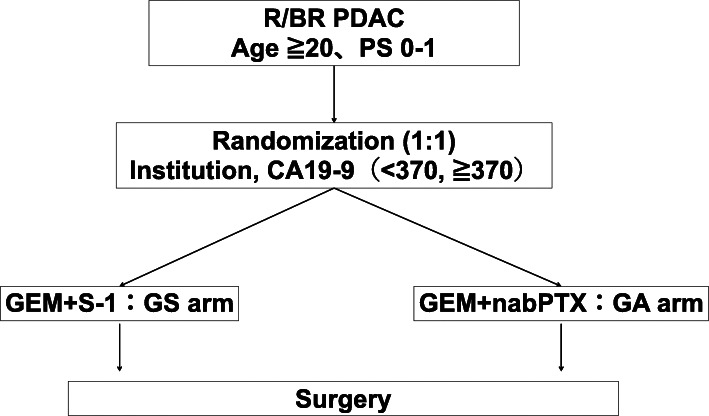
Schematic flowchart of the CSGO-HBP-015 study. R, resectable; BR, borderline resectable; PDAC, pancreatic ductal adenocarcinoma; PS, performance status according to the Eastern Cooperative Oncology Group

CSGO-HBP-015 is a multicenter, two-arm, open-label, randomized, exploratory trial with two treatment arms allocated in a 1:1 ratio (Fig. 1). Eligible patients were centrally registered at a nonprofit organization, the Supporting Center for Clinical Research and Education (SCCRE), Osaka, Japan. Block randomization was by a computer-generated random number list prepared by a staff of SCCRE with no clinical involvement in the trial, and the allocation sequence was concealed from the researchers.

## Methods: participants, interventions, and outcomes

### Study setting {9}

This trial is an intergroup cooperative study led by the Clinical Study Group of Osaka University, Hepato-Biliary Pancreatic Group (CSGO-HBP), CSGO-HBP-015, with participating institutions including 17 specialized centers on 30 March 2016.

### Eligibility criteria {10}

#### Inclusion criteria

- Treatment-naïve PDAC patients with a histological or cytological diagnosis, including adenocarcinoma and adenosquamous carcinoma
- Age ≥ 20 years
- Eastern Cooperative Oncology Group (ECOG) performance status of 0 or 1
- R0/1 resectable*, localized tumor without distant metastasis (liver, peritoneum, lung, or others) confirmed by radiological evaluation (enhanced computed tomography (CT))

*A resectable tumor is defined as follows:

- The tumor does not contact any major vessels (e.g., superior mesenteric vein (SMV), portal vein (PV), common hepatic artery (CHA), or celiac artery (CA))
- The tumor is suspected to contact/involve the SMV or PV but there are suitable vessels proximal and distal to the site of involvement, allowing a safe and complete resection and vein reconstruction
- The tumor is in contact with other organs (e.g., stomach or colon) but allows a safe and complete resection
- The tumor is in contact with the inferior vena cava (IVC)
- The tumor (located in the pancreatic body or tail) is in contact arterial abutments, including the CHA and/or CA, but allows a safe and complete resection without arterial reconstruction by modified Appleby surgery (i.e., distal pancreatectomy with celiac axis resection (DP-CAR))
- Life expectancy of more than 6 months
- Spared organ function satisfying the following laboratory data: white blood cell count of neutrophils ≥ 1500/mm^3^, platelet count ≥ 100 000/mm^3^, hemoglobin ≥ 9.0 g/dl, serum total bilirubin ≤ 2.0 mg/dl, aspartate aminotransferase (AST) ≤ 150 IU/l, alanine aminotransferase (ALT) ≤ 150 IU/l, total bilirubin ≤ 2.0 mg/dl (or ≤ 3.0 mg/dl if biliary drainage was present), creatinine ≤ 1.2 mg/dl, and creatinine clearance ≥ 60 ml/min
- No smoking history or mild smoking status based on the Brinkman index (the number of cigarettes smoked per day multiplied by the number of years of smoking) ≤ 200*

*The inclusion criteria concerning smoking status have been included since September 2017

- Written, informed consent

#### Exclusion criteria

- Unresectable tumor, which is defined as follows:
- The tumor is accompanied by distant metastasis (liver, peritoneum, lung, para-aorta lymph nodes, or others) on imaging
- When suspected, staging laparoscopy is performed. If peritoneal dissemination is detected or cytologic examination of ascitic fluid is positive, the case is categorized into the unresectable stage
- The tumor involves major vessels, including the abdominal aorta, CA, CHA, or primary hepatic artery, and does not allow complete resection without arterial reconstruction
- The tumor is accompanied by unreconstructible SMV/PV involvement
- Pulmonary fibrosis or intestinal pneumonia (anamnesis or imaging findings)
- Severe diarrhea
- Active infection
- Severe complications (e.g., heart failure, renal failure, hepatic insufficiency, hemorrhagic peptic ulcer, intestinal paralysis, ileus, or uncontrolled diabetes)
- Massive pleural or abdominal effusion
- Active synchronous malignancy except for carcinoma in situ or intramucosal tumor after adequate curative treatment
- Metachronous malignancies except for diseases with relapse-free survival ≥ 3 years
- Regular use of frucitocin, phenytoin, or warfarin
- Pregnancy, breastfeeding, or desire of a woman to preserve fertility
- Severe mental illness
- Severe allergies to drugs
- Patients inappropriate for this study as judged by the treating physician

### Who will take informed consent? {26a}

The treating physician in each hospital will obtain informed consent from each eligible patient. After an explanation and after reading the patient information sheet, the patients are asked to consider participation. If necessary, further explanation will be provided.

### Interventions {11a}

#### Treatment

##### Neoadjuvant chemotherapy

**Fig. 2:**
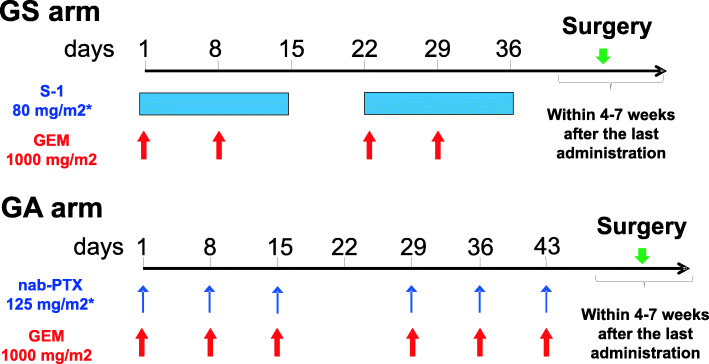
Schematic time schedule of both neoadjuvant chemotherapy arms. GS, gemcitabine and S-1 regimen; GEM, gemcitabine; GA, gemcitabine and nab-paclitaxel regimen. *Dose density is according to the body surface area (BSA). BSA < 1.25 m^2^, 80 mg/day; 1.25 ≤ BSA < 1.5 m^2^, 100 mg/day; BSA ≥ 1.5 m^2^ 120 mg/day

Patients allocated to the GS arm will receive intravenous gemcitabine at a dose of 1000 mg/m^2^ on days 1 and 8 plus S-1 orally at a dose according to their body surface area (BSA) (BSA < 1.25 m^2^, 40 mg; BSA 1.25–1.5 m^2^, 50 mg; BSA > 1.50 m^2^, 60 mg) twice daily on days 1–14 of a 21-day cycle (Fig. 2). Patients with a creatinine clearance of 50–60 ml/min will receive a dose of S-1 that is reduced by 20 mg/day.

Patients allocated to the GA arm will receive intravenous GEM and subsequent nab-paclitaxel (nab-PTX) at doses of 1000 mg/m^2^ and 125 mg/m^2^, respectively, according to their BSA on days 1, 8, and 15 of a 28-day cycle (Fig. 2). For patients receiving GA treatment, if patients develop grade 3–4 neutropenia, grade 2–4 thrombocytopenia, or grade 2–4 nonhematological toxicity, both gemcitabine and nab-PTX will be withheld until recovery or reduced at treatment resumption (GEM −200 mg/m^2^/day and nab-PTX −25 mg/m^2^/day), according to the guidelines for Japanese patients.

These neoadjuvant treatments are repeated for two cycles unless unacceptable toxicity, such as grade 4 adverse events evaluated by the Common Terminology Criteria for Adverse Events (CTCAE, version 4.0), occurs.

Restaging by CT is required before surgery. In cases of unexpected tumor progression (unresectable tumor extension or distant metastasis), the patients will receive palliative treatment, including chemotherapy and/or radiotherapy, as off-protocol care.

##### Surgery

Patients who receive either NAC treatment will undergo surgery within 4–7 weeks after the last administration of chemotherapy (oral S-1 or intravenous GEM/nab-PTX). Depending on the individual tumor site and its extension, patients in both study arms will undergo curative-intent pancreatectomy with regional node dissection. Intraoperative peritoneal lavage cytology is required. In cases of unexpected intraoperative findings regarding unresectability, including distant metastasis or inseparable tumor extension into major arteries (HA, CA, or SMA), patients will not undergo resection but will undergo a suitable bypass procedure if necessary.

##### Adjuvant chemotherapy

Although this study does not stipulate adjuvant chemotherapy after surgery, adjuvant therapy after macroscopically curative resection with histologic R0 or R1 residual disease is highly recommended. Patients usually receive S-1 (otherwise GEM) as adjuvant chemotherapy after surgery for 6 months. Patients ineligible for adjuvant chemotherapy receive palliative treatment and are observed.

### Criteria for discontinuing or modifying allocated interventions {11b}

The dosage of chemotherapy drugs in each regimen will be modified according to the guidelines when the patients show adverse events due to potential complications of the chemotherapy drugs.

When the following criteria are met, further treatment is discontinued.

1. Patients withdraw their consent
2. Tumor progression
3. Situations in which the treating physician deems it is difficult to continue the protocol treatments (e.g., adverse events due to protocol treatments, house moving, or severe comorbidities)

### Strategies to improve adherence to interventions {11c}

Adherence to the intervention protocol is maximized by carefully counseling the participants, training the study staff, and monitoring using drug accountability logs. If necessary, training is repeated during the course of the study. All physicians involved in clinical trials in Japan will undergo good clinical practice (GCP) training and protocol training.

### Relevant concomitant care permitted or prohibited during the trial {11d}

The use of frucitocin, phenytoin, or warfarin is an exclusion criterion. Changes in other comedications during the study are allowed, as this is not expected to influence the disease course. The administration of other anticancer drugs is contraindicated in patients. Granulocyte colony-stimulating factor (G-CSF) can be used to treat severe neutropenia.

### Provisions for posttrial care {30}

The patients will be followed up for 5 years after the completion of patient accrual. To investigate recurrence, three types of examinations will be performed every 3–4 months for 5 years: a routine physical examination; laboratory tests, including the analysis of the serum level of CA19-9 (tumor marker); and radiological imaging, including chest and abdominal CT (or MRI). The date of recurrence is defined as the date that the investigator detects recurrence on an image or in a biopsy specimen. Toxicities will be evaluated according to the CTCAE, version 4.0. This study is conducted under the standard health insurance treatment, and patients that are enrolled into the study are covered against adverse events through the standard National Health Insurance.

### Outcomes {12}

#### Primary endpoint

The primary endpoint of this study is PFS. PFS is calculated from the day of randomization to the day of death from any cause and is censored on the last day that the patient is documented to be alive without tumor progression. Tumor progression is defined as the appearance of a new lesion on the image or according to the surgeon’s findings during surgery. If the growth of the primary lesion expanding to an unresectable lesion is detected before surgery, the tumor is assumed to have progressed. Detecting any recurrence site is considered tumor progression after surgery. Information on tumor progression types should be collected to evaluate each rate.

#### Secondary endpoints

The secondary endpoints are the curative resection rate, dose intensity, radiological/histological responses for both NAC arms, recurrence type (if developed), OS, and adverse events. The curative resection rate is defined as the proportion of resected cases without histological residual tumor after either NAC treatment. OS is calculated from the day of randomization to the day of death from any cause and is censored on the last day that the patient is documented to be alive.

### Participant timeline {13}

**Fig. 3:**
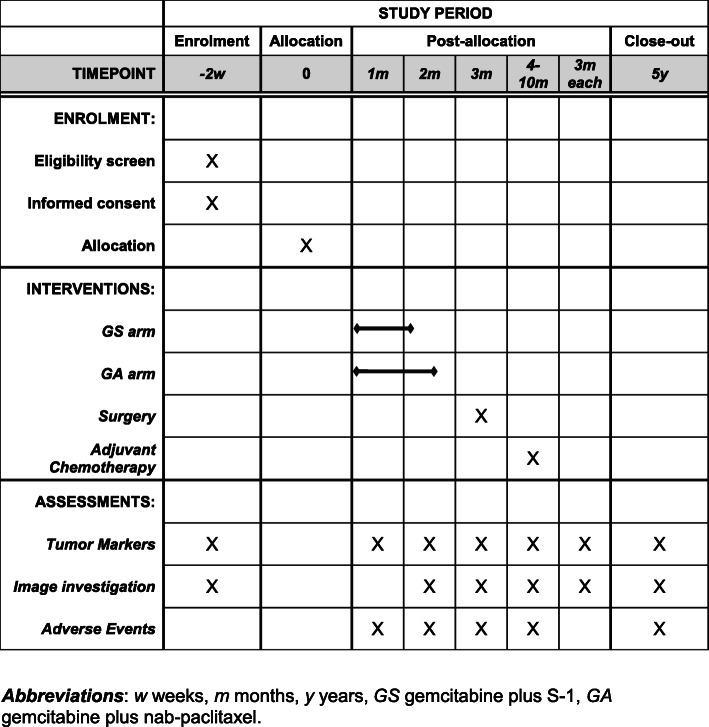
Participant timeline. *Abbreviations*: *w* weeks, *m* months, *y* years, *GS* gemcitabine plus S-1, *GA* gemcitabine plus nab-paclitaxel

After the intervention period, patients will be followed for another 5 years. The choice of treatment in the case of relapse is at the discretion of the treating physician. The treatment protocol is illustrated in Fig. 2, and the time schedule of enrollment, interventions, and assessments is exhibited in Fig. 3.

### Sample size {14}

This study is designed as a randomized phase II trial to examine the efficacy, in terms of PFS, of both NAC regimens (GA and GS) followed by surgery in patients with PDAC for whom surgery is planned. In this clinical trial, the clinical hypothesis is that the NAC treatment, GS or GA, with superior PFS is a more favorable NAC regimen for patients with resectable PDAC. The survival data of the arm analyzed by intention-to-treat have been limited to meet assumptions. In this study, if PDAC patients in a similar stage receive a standard treatment consisting of surgery followed by adjuvant chemotherapy, the 2-year survival probability of patients is estimated to be 50–60% with the following concepts: (1) approximately 80% of patients can undergo curative surgery after NAC [6, 7, 20], (2) 70% of patients can complete adjuvant chemotherapy after curative surgery [21], (3) the 2-year survival rate of patients completing adjuvant chemotherapy following curative surgery is 70% [4, 21, 22], and (4) the 2-year survival rate of patients not completing adjuvant chemotherapy or curative surgery is 40% [4, 21, 22]. We assumed that NAC does not impair the completion rate of adjuvant chemotherapy or OS [6, 7, 20, 21], and thus, the 2-year PFS probability of patients with NAC is expected to be at least 60%. In summary, a surrogate hypothesis was established to calculate the sample size as follows: the 2-year PFS of patients with inferior NAC treatment is 60%, and we can select the superior NAC treatment if the 2-year PFS of patients with the superior NAC treatment is over 70%. For the 2 arms with inferior PFS = 60% and superior PFS = 70%, to have at least 80%, 85%, and 90% probabilities of selecting the better arm, we need sample sizes (*n*) of 33, 50, and 76 patients per arm, respectively. The planned total sample size is 100, with a power of 80–85%.

### Recruitment {15}

The average number of surgeries for patients with localized PDAC is approximately 200 per year in our institutions (CSGO-HBP). We assume that 15–20% of them will meet the inclusion criteria; thus, we expect to reach the target sample size within 4 years from the start of the trial.

### Assignment of interventions: allocation

Eligible patients were centrally registered and randomly assigned to treatment at SCCRE. The investigators were masked to the randomization, which was performed by the block randomization method. The patients were stratified according to the institution and serum CA19-9 value (< 370 ml vs ≥ 370 U/ml). The serum CA19-9 value must be measured without jaundice or after adequate biliary drainage (serum total bilirubin ≤ 3 mg/dl). Eligible patients were randomized (1:1) to receive either NAC regimen (Fig. 1).

### Data collection and management

#### Plans for assessment and collection of outcomes {18a}

The treating physicians report outcomes on a set case report form (CRF) and send them to the SCCRE. The principal investigators (PIs) organize and analyze the collected data from the CRFs.

#### Plans to promote participant retention and complete follow-up {18b}

Patients who reach a preliminary endpoint or drop out of the study for any reason will be treated at the physician’s discretion and will complete follow-up according to the protocol. If patients are unable to complete follow-up, the data obtained up until that point will be stored in a CRF and used in the analysis if appropriate.

#### Data management {19}

Source documents for each patient will be kept at the patient’s study site until the last follow-up visit of the last patient included. Records will include the date of informed consent, medical history, and study parameters. Data are recorded on the CRF. After the end of the study, individual patient data will be stored at the SCCRE. After the end of the study, all essential forms pertaining to the conduct of the study will be archived by the investigator for a period of 5 years. The coordinating PI is the only one who has access to this information. All patient data are stored in a way that the privacy of participants is respected. Data validation procedures (such as reason for change logs, value and range checks, prompts and warnings if data are entered incorrectly) are built into the database to promote data quality. An audit trail is in place. Both a monitoring plan and a data management plan are in place to promote data quality.

#### Confidentiality {27}

Each patient is labeled with a trial number (consecutive patient number), and the data are organized with that number. Only the local investigators will have access to the trial number of patients treated at the study site. As the data collected for this study are collected in normal medical care (e.g., tumor markers, image data, and tumor information), they do not contain special information for the local investigator.

#### Plans for collection, laboratory evaluation, and storage of biological specimens for genetic or molecular analysis in this trial/future use {33}

No biological specimens for genetic or molecular analysis will be collected during this trial.

### Statistical methods

The primary endpoint of PFS will be based on the intent-to-treat (ITT) population, which includes all eligible patients enrolled in the study. Both PFS and OS (secondary endpoint) will be additionally analyzed on a per-protocol (PP) basis. Kaplan–Meier analysis and the log-rank test will be used to construct survival curves and to evaluate differences in the univariate analysis for PFS and OS. For the comparison of the other outcomes, the chi-squared test (for the curative resection rate, radiological/histological responses, recurrence type (if developed), and adverse events) and Fisher’s exact test (for dose intensity) will be used to compare categorical variables. Logistic regression will be performed for both the multivariate analysis and the partition analysis of the detected factors in univariate analysis.

Interim efficacy analyses will not be performed.

We do not plan to perform additional analyses so far.

### Methods in analysis to handle protocol nonadherence and any statistical methods to handle missing data {20c}

In the ITT analysis, all randomized patients will be analyzed in the treatment group to which they were originally allocated, irrespective of potential eligibility deviation, nonadherence, or other deviations from the protocol. In the PP analysis, patients who were included in accordance with the study protocol and who completed the treatment originally allocated will be analyzed. Patients who deviated from the protocol will be excluded from the per-protocol population. Patients withdrawing consent after randomization, but prior to the start of the treatment protocol, will be excluded from the analysis.

### Plans to give access to the full protocol, participant-level data, and statistical code {31c}

The protocol and generated datasets, including the statistical code, are available from the corresponding author upon reasonable request.

### Oversight and monitoring

#### Composition of the coordinating center and trial steering committee {5d}

The Department of Gastroenterological Surgery of Osaka University is responsible for trial management. All participants’ hospitals (17 institutions, list on page 1) in CSGO-HBP recruited and treated eligible patients and collected data. The trial steering committee is CSGO-HBP, consisting of 51 institutions, and representatives in these institutions meet every 3 months for the trial. The SCCRE organizes and manages the data collection.

#### Composition of the data monitoring committee, its role, and reporting structure {21a}

The monitoring and quality assurance of the study are performed by centralized monitoring in compliance with GCP. The PI, corresponding investigator, and Data and Safety Monitoring Board (DSMB), consisting of two staff members (H. Nagano from the Department of Gastroenterological, Breast and Endocrine Surgery of Yamaguchi University and S. Marubashi from the Department of Hepato-Biliary-Pancreatic and Transplant Surgery of Fukushima Medical University), perform regular monitoring once a year. A review of the submitted data for quality is performed to identify and address missing and inconsistent data. Monitoring visits are not performed, but additional information can be collected from each institution if necessary. The data collected throughout the study are monitored and checked for accuracy and completeness. Safety reporting, including adverse event reports and their proper administrative handling, will be checked for accuracy and completeness.

### Adverse event reporting and harms {22}

Adverse events are defined as any undesirable experience occurring to a patient during treatment, including NAC, surgery, and adjuvant chemotherapy, whether or not considered related to the study drugs (GA, GS). All adverse events reported spontaneously by the patient or observed by the investigator or his staff are recorded during the treatment period. Severe adverse events that result in death or are life threatening will be reported within 7 days or 15 days. Severe adverse events will also be reported according to the national and local regulations if necessary. All adverse events will be followed until they have abated or until a stable situation has been reached. Depending on the event, follow-up may require additional tests or medical procedures as indicated and/or referral to the general physician or a medical specialist.

### Frequency and plans for auditing trial conduct {23}

There are no plans for an audit of trial conduct, but it will be performed if required.

### Plans for communicating important protocol amendments to relevant parties (e.g., trial participants, ethical committees) {25}

All amendments will be submitted for approval to the Osaka University Clinical Research Review Committee. Relevant changes to the protocol and/or study management are reported to the study staff and, if applicable, to the participants. The trial registry will be updated following substantial amendments. The PI will submit a summary of the progress of the trial to the Osaka University Clinical Research Review Committee once a year. Information on the date of inclusion of the first patient, numbers of patients included and numbers of patients who have completed the trial, severe adverse events, other issues, and amendments will be provided.

### Dissemination plans {31a}

The trial results will be published in a peer-reviewed journal (e.g., *Annals of Surgery*, *JAMA Surgery*, etc.) and presented at local (e.g., annual congress of Japan Surgical Society, general meeting of the Japan Society of Gastroenterological Surgery) and international conferences (e.g., American Society of Clinical Oncology: Gastrointestinal Cancers Symposium). All participants who wish to know the study results will also be contacted directly regarding their published results.

## Discussion

This study is the first randomized phase II study comparing GS combination therapy with GA combination therapy as NAC for localized PDAC. The trial was designed with two kinds of NAC regimens added to the present standard treatment consisting of surgery followed by adjuvant chemotherapy, and it will convey noteworthy results concerning whether one regimen is more preferable than the other regimen in the following standpoints: positive effects on survival, safety, and feasibility during treatment, including NAC, surgery and adjuvant chemotherapy, resection rate, and radiological/histological responses. These findings will contribute to selecting the optimal NAC regimen for patients with localized PDAC.

Moreover, the cumulative safety information is significant. The key chemotherapy drug GEM is well tolerated; however, the administration of GEM induces pulmonary toxicity as a severe adverse event with a reported rate of 0.1–2.5% [23]. Severe pulmonary toxicity associated with chemotherapy drugs typically presents as interstitial lung disease (ILD), in which the parenchymal or alveolar regions are affected by inflammation and fibrosis. The chemotherapy drug nab-PTX also induces ILD with a reported rate of 1.5%, and the combination therapy of the GA regimen possibly increases the rate of ILD with a reported rate of 2.2–19.2% [17, 23–25]. In this trial, one patient in the GA arm developed acute and severe ILD during NAC treatment. The case was immediately reported to the DSMB, and we stopped enrollment for 9 months from January 2017 to September 2017. The patient had a severe smoking history, and the development of ILD due to the administration of the GA regimen was possibly associated with this severe smoking history [23]. Thus, the CSGO-HBP and DSMB decided to add the new inclusion criterion concerning smoking history. To allow the registration of patients with a negligible smoking history but not the registration of patients with a severe smoking history, patients with no smoking history or a mild smoking status based on the Brinkman index (the number of cigarettes smoked per day multiplied by the number of years of smoking) ≤ 200 are now eligible. After restarting enrollment, severe adverse events of ILD have not been reported.

When this trial is completed, we believe that it may provide prospective data on the superiority of one NAC regimen over the other NAC regimen for patients with localized PDAC from various standpoints.

## Trial status

The first participant was enrolled on 26 April 2016. There are currently 93 patients enrolled out of 100, and the estimated completion of recruitment is the end of 2021. The current version of the protocol is 4.0, dated 4 December 2020.

## Data Availability

The datasets used and/or analyzed during the current study are available from the corresponding author on reasonable request.

## Abbreviations

PDAC: Pancreatic ductal adenocarcinoma
R: Resectable
BR: Borderline resectable
NCCN: National Comprehensive Cancer Network
NAC: Neoadjuvant chemotherapy
GEM: Gemcitabine
OS: Overall survival
GS: Gemcitabine plus S-1
GA: Gemcitabine plus nab-paclitaxel
PFS: Progression-free survival
CSGO-HBP: Clinical Study Group of Osaka University, Hepato-Biliary Pancreatic Group
ECOG: Eastern Cooperative Oncology Group
CT: Computed tomography
SMV: Superior mesenteric vein
PV: Portal vein
CHA: Common hepatic artery
CA: Celiac artery
IVC: Inferior vena cava
AST: Aspartate aminotransferase
ALT: Alanine aminotransferase
BSA: Body surface area
nab-PTX: Nab-paclitaxel
CTCAE: Common Terminology Criteria for Adverse Events
GCP: Good Clinical Practice
G-CSF: Granulocyte colony-stimulating factor
SCCRE: Supporting Center for Clinical Research and Education
CRF: Case report form
PI: Principal investigator
ITT: Intent-to-treat
PP: Per-protocol
DSMB: Data and Safety Monitoring Board
